# The Current State of Radiomics for Meningiomas: Promises and Challenges

**DOI:** 10.3389/fonc.2020.567736

**Published:** 2020-10-27

**Authors:** Hao Gu, Xu Zhang, Paolo di Russo, Xiaochun Zhao, Tao Xu

**Affiliations:** ^1^ Department of Neurosurgery, Changzheng Hospital, Naval Medical University, Shanghai, China; ^2^ Department of Neurosurgery, I.R.C.C.S. Neuromed, Pozzilli, Italy; ^3^ Department of Neurosurgery, University of Oklahoma Health Sciences Center, Oklahoma City, OK, United States

**Keywords:** meningioma, radiomics, medical imaging, diagnosis, deep learning

## Abstract

Meningiomas are the most common primary tumors of the central nervous system. Given the fact that the majority of meningiomas are benign, the preoperative risk stratification and treatment strategy decision-making highly rely on the conventional subjective radiologic evaluation. However, this traditional diagnostic and treatment modality may not be effective in patients with aggressive-growing tumors or symptomatic patients with potential risk of recurrence after surgical resection or radiotherapy, as this passive “wait and see” strategy could miss the optimal opportunity of intervention. Radiomics, a new rising discipline, translates high-dimensional image information into abundant mathematical data by multiple computational algorithms. It provides an objective and quantitative approach to interpret the imaging data, rather than the subjective and qualitative interpretation from relatively limited human visual observation. In fact, the enormous amount of information generated by radiomics analyses provides radiological to histopathological tumor information, which are visually imperceptible, and offers technological basis to its applications amid diagnosis, treatment, and prognosis. Here, we review the latest advancements of radiomics and its applications in the prediction of the pathological grade, pathological subtype, recurrence possibility, and differential diagnosis of meningiomas, and the potential and challenges in general clinical applications. In this review, we highlight the generalization of shared radiomic features among different studies and compare different performances of popular algorithms. At last, we discuss several possible aspects of challenges and future directions in the development of radiomic applications in meningiomas.

## Introduction

Meningiomas are the most common primary central nervous system (CNS) tumors, composing up to 36.4% of all CNS tumors, with an incidence of 7.86/100000 (1). The majority of meningiomas are benign (2), while only 1% are malignant (1) but with increased morbidity and mortality rates (3). According to the 2016 edition of the WHO classification of CNS tumors, meningiomas are considered as heterogeneous tumors that can be divided into three grades and 15 different pathological subtypes (4). Moreover, meningiomas may present also an intratumoral heterogeneity, such as different degrees of growing patterns, vascularization, necrosis, infiltration, etc., in the same tumor. The transformation from low-grade to high-grade meningiomas is a rare event that can happen as consequences of this intratumoral heterogeneity (5). These intertumoral and intratumoral heterogeneities can explain the different outcomes after resection of meningiomas. Thus, better understanding of the actual biological behavior of these tumors preoperatively could benefit the risk stratification and decision-making process. For example, the “wait and see” modality with a longer imaging follow-up period would be an ideal and cost-effective option in small, stable and benign meningiomas (6). On the other hand, early surgical resection should be recommended in patients with meningiomas which are small in size, but active in growing, or malignant in genotyping.

Nowadays, medical imaging plays a fundamental role in the process of preoperative and differential diagnosis in the CNS tumors such as meningiomas (7). Modern imaging technology, as 3T MRI, provides sufficient high-quality information of the lesions, such as post contrast T1-weighted images can highlight enhancing regions within the tumor because of the leakage of contrast agent from the intravascular lumen into the tumor through a disrupted blood-brain barrier (8), or the integrated use of fluid-attenuated inversion-recovery sequences and T2-weighted images to delineate a more precise boundary between edema and the solid tumor (9). Nevertheless, these data are commonly reported by the radiologist in a descriptive, qualitative, and subjective way. As a dural-based lesion, meningiomas can be misdiagnosed, especially when the radiologists are not familiar with the differential diagnosis of other dural-based lesions. For example, the “dural tail sign” on enhanced T1-weighted sequences, a characteristic imaging sign often regarded as the representative of meningiomas (10), can also be positive in other diseases including sarcoidosis (11), lymphoma (12), metastases (13–15) and other lesions (16, 17). Also, some imaging features, such as the peritumoral edema and morphological irregularity of the meningioma, which may suggest an aggressive pattern, have not been validated yet (18, 19).

Recently, some promising progresses in the preoperative diagnosis have emerged in the field of oncology, as well as in meningiomas. In this scenario, radiomics analysis refers to different methods that “decode” the quantitative features of medical images across different types of tumors. The primary intention of this technique is to identify, from radiological images, several quantitative characteristics of the tumor, so they can be used to improve the understanding of the pathology and biology of the lesion. This data are also sought to predict clinical outcomes, such as patients’ survival and responses to therapy (7). The features commonly included in this type of analysis are: volume, shape, intensity (MRI signal) and other texture features, referring to pixel intensities, their distribution pattern, and their interrelationships (20). Nowadays, the radiomics analysis has been used for various types of cancer including lung cancer (21–23) and prostate cancer (24–26). Yet, only few studies reported on radiomics analysis of meningiomas. This analysis can help in preoperative diagnosis by adding new information, such as the growth rate of an incidental meningioma, guiding the differential diagnosis of tumors with dural implantation, predicting tumors’ recurrence, and subsequently tailoring the treatment strategies. In this study, we discuss the latest application of radiomics analysis for meningiomas and the potential clinical implications of its integration in preoperative diagnosis.

## Overview of the Workflow of Radiomics

**Figure 1:**
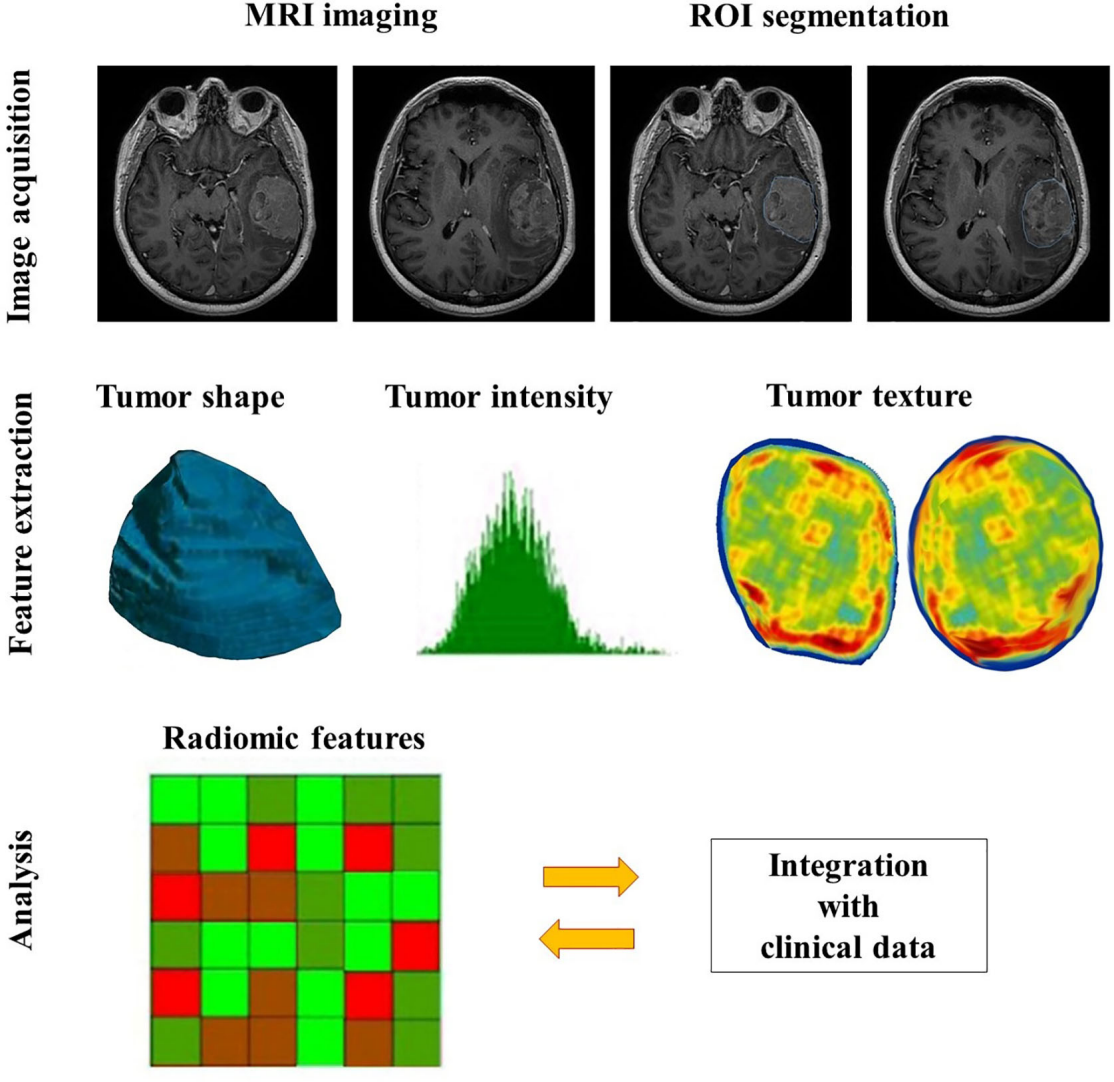
The general workflow of radiomics in meningiomas includes image acquisition, ROI segmentation, feature extraction and analysis.

Generally, the procedure of radiomics can be divided into four main steps (7, 27, 28): image acquisition, segmentation, feature extraction, and statistical analysis/model (**Figure 1**), but each step is somewhat different across various studies for different purposes (29).

### Image Acquisition

Image acquisition is the first step of the radiomics workflow, including acquisition and reconstruction of the image data (29). Acquiring image data refers to collecting raw data with full annotations of multiple imaging parameters, such as repetition time, echo time and field of view in the MRI images, tube voltage and tube current in the CT images, etc., which can be extracted from the image software (28). Image reconstruction is the process of transforming the raw non-image-formative data into the image format by various algorithms. Because the variations in the imaging scanners, modalities, sequences, parameters, and reconstruction algorithms are likely to impact on the results of the final analysis, it is necessary to provide quantitative imaging with error bars or standardizing the original image data to improve the homogeneity (29).

### Segmentation

The second step contains identification and segmentation of the region of interest (ROI), either manually, automatically or semi-automatically. In clinical settings, meningiomas are usually manually delineated by experienced radiologists. Given the fact that many other types of tumor do not have distinct borders and their inside heterogeneity, the accompanying inter-user variability is an inevitable issue. There are several strategies to minimize the variability, the common one is the segmentation tools (28). The rational choice of segmentation software and double-check with vision manually can not only optimize the result but also raise the efficiency of workflow, especially when a radiologist handles hundreds of cases at the same time. Other approaches such as application of an algorithm (30) or segmenting a fixed-size ROI (31) also work in certain scenarios.

### Feature Extraction

Feature extraction is to decode the high-dimension image data and output them quantitatively (29). In the present, the patterns of feature extraction can be simply classified into with or without human orders (7). The conventional way needs specialized algorithms under human instructions. While the newer mode can nearly complete the rest of the whole task automatically and independently from human aids, which is based on the deep learning radiomics (DLR), such as convolutional neural networks (CNNs). Moreover, the number of extracted features within the CNNs is several orders of magnitude greater than the conventional methods (7), but it is necessary to reduce the feature dimensions to avoid overfitting (29). Besides, feature extraction, selection, and classification can occur across different layers in the same CNN (7).

Features in radiomics are divided into two groups, semantic and agnostic. Semantic features indicate the radiology lexicons which are commonly used to intuitively describe the lesion, such as size, location, and shape. Conversely, agnostic features are mathematically-extracted quantitative descriptors, which aim to highlight the lesion heterogeneity (29). Agnostic features can be subdivided into three categories, which are first-, second-, and higher-order. First-order statistics depict the distribution of values of individual voxels without any concerns of the spatial relationships, mostly based on the histogram, such as skewness and kurtosis. Second-order statistics describe statistical interrelationships between voxels with similar (or dissimilar) contrast values, termed as “texture” features. Higher-order statistical features are repetitive or nonrepetitive patterns filtered through specific grids on the image, for example, Laplacian transforms, Minkowski functionals, etc. (29).

### Statistical Analysis/Modeling

In the final step, the selected features can be used for many different analyses, and they are mostly incorporated into predictive models to provide improved risk stratification (28). Model construction is the process of developing integration of a set of analysis methods, involving with clustering features and assigning these features with different values according to the predefined information content. Those analysis approaches include artificial intelligence, machine learning, and statistical methods. An ideal model can not only handle the extracted features adequately, but also is able to accommodate sparse data, for instance, genomic profiles (29). The more covariates it can handle, the more specific meaning of a model can be.

However, it would be rather difficult for an inexperienced user to make a choice among multiple algorithms for model building. This situation promotes the implementations of multiple-modelling methodology in a single study, although it may not be necessary (32). The fundamental principle of selecting an algorithm is the reproducibility of the whole process (32), which could be enhanced by a set of measures: (I) evaluate the feature reproducibility; (II) conduct the cross-correlation analysis; (III) contain clinically significant variables (volume included); (IV) warrant sufficient observation rates (at least 10–15 per feature); (V) provide an external validation cohort; (VI) interpret radiomic features of no physical (or biological) meaning with prudency (33).

## Current Application of Radiomics in Meningiomas

**Figure 2:**
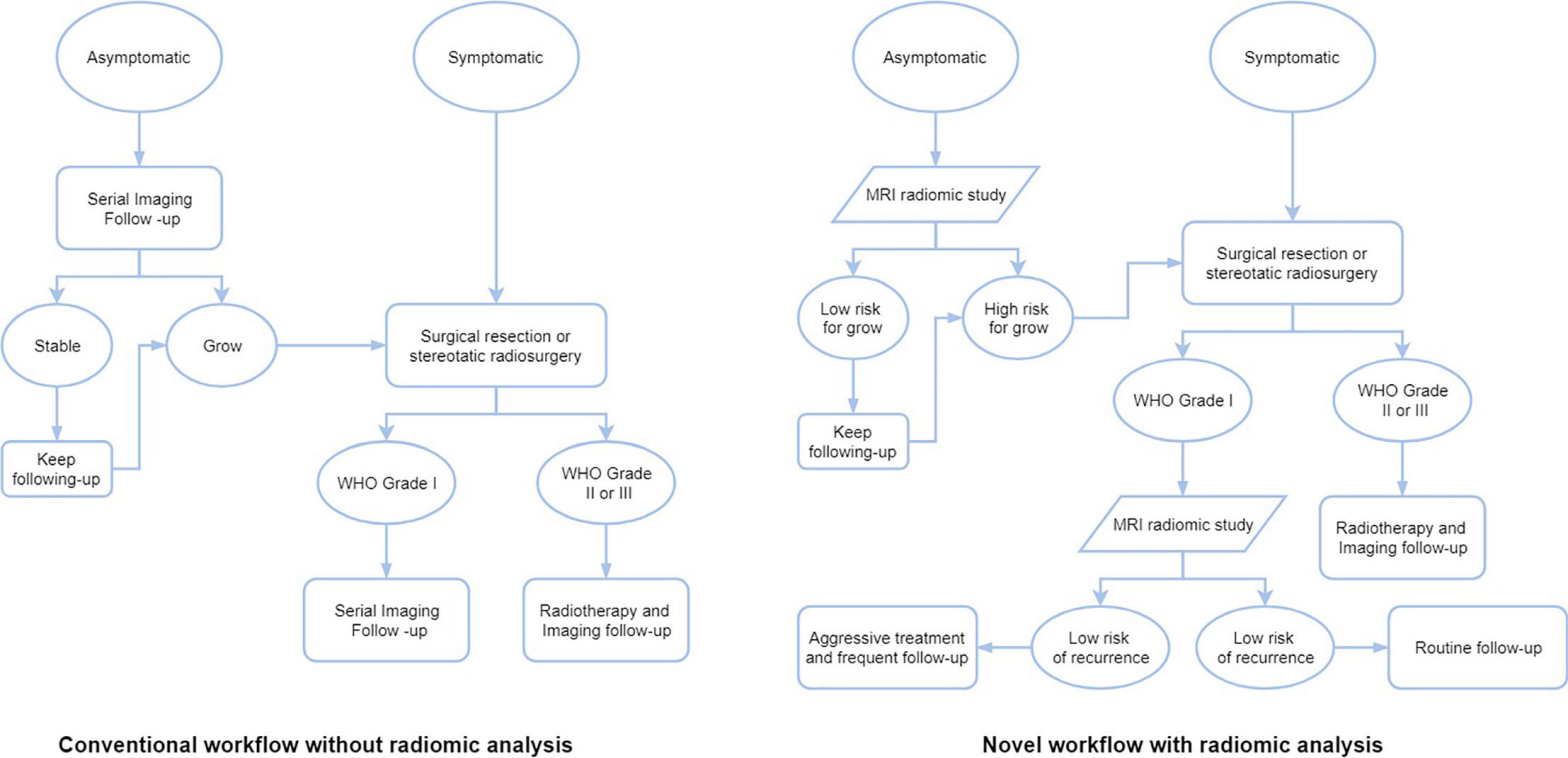
The workflows of different treatment strategies of meningiomas without or with radiomic analysis.

Most studies using radiomic analysis in meningioma were based on the MRI, ranging from a single to multiple imaging sequences. Indeed, the MRI can provide a superior anatomical delineation (e.g. spatial location) of the intracranial structures and characterize the predominance of different physiopathological processes, due to the different sensitivity of tumor physiology in various MRI imaging sequences (8). In general, the application of radiomics in meningioma can be roughly divided into two aspects: grade prediction and other applications (**Table 1**). The workflow of treating meningiomas may alter based on these radiomic findings (**Figure 2**).

**Table 1:** Summary of previous reported application of radiomics in meningiomas.

Research groups	Sample Size	Modality	Aim	Segmentation	Features	Feature Selection	Statistical Analysis	Result	Conclusion
Coroller et al. (34)	175	TICE	Grading	Manual	Intensity, Histogram, Texture, Wavelet, LoG	PCA FA	RF	Validation: AUC = 0.78, P = <0.0001. Radiomic classifier could significantly predict meningioma grade.	Demonstrated the ability to discriminate between WHO grade I from grade II/III, which was ready for clinical application.
Yan et al. (35)	131	T1WI+C, T2WI, FLAIR	Grading	Manual	Histogram, Gradient, RLM, CM, Wavelet transform, Autoregressive model	CfsSubset-Eval evaluator in the data mining software Wek	LR, NB, SVM	Training: AUC = 0.87, P = <0.0001. The SVM classifier built on all six representative radiomic features achieved the best performance, with a sensitivity, specificity, diagnostic accuracy of 0.86, 0.87, 0.87, respectively.	Demonstrated that a SVM classifier based on texture and shape features was able to satisfactorily predict meningioma grade before surgery.
Laukamp et al. (36)	71	T1WI, T2WI, T1-CE, FLAIR, DWI/ADC	Grading	Semiautomatic	Shape, Texture	RF	Multivariate logistic regression analysis	AUC = 0.91, P = <0.001. A radiomic classifier built on the combination of the features was able to differentiate grade I and grade II meningiomas.	Demonstrated the ability to discriminate between WHO grade I from grade II, and a classifier built on the combined features had the best performance.
Hamerla et al. (37)	138	T1WI, T2WI, T1-CE, Sub, FLAIR, ADC,	Grading	Semiautomatic	Shape, Histogram, Texture, A set of high-order statistical features	Mann-Whitney U test	RF, XGBOOST, SVM, MLP	Validation: AUC = 0.97, sensitivity = 1.0, specificity = 0.97 The best classifier was XGBoost consisting of the combination of ADC, ADC of the peritumoral edema, T1, T1c, Sub and FLAIR-derived features.	Demonstrated the ability to classify meningiomas between WHO grade I and grade II/III despite the heterogeneity of raw imaging data from different centers by the method of machine learning.
Chen et al. (38)	150	TIWI, T1CE	Grading	Manual	Histogram, Shape, Texture, High-order features	Distance correlation, LASSO, GBDT	LDA, SVM	Validation: Accuracy=0.756, Kappa value=0.603. The Lasso + LDA model represented the highest diagnostic performances than SVM-based models.	Demonstrated that machine learning algorithms with texture features extracted from T1C images could preoperatively classify meningioma grades.
Zhu et al. (39)	118	T1CE	Grading	Manual	Deep learning features	RF, SBS	LDA	Primary: AUC = 0.891, P = <0.001. Validation: AUC = 0.811, P = <0.001. DLR features could function as effective grade predictor, and their performance was better than the hand-crafted features.	Demonstrated that a DLR model was able to discriminate between WHO grade I from grade II/III, and its capacity was stronger than HCR model.
Park et al. (40)	136	TICE, ADC, FA	Grading, Subtyping	Semiautomatic	Histogram, Texture	Recursive feature elimination	SVM, RF	Validation: AUC = 0.86, accuracy=0.897, sensitivity = 0.75, specificity = 0.935. The machine learning classifiers showed different performances according to the machine learning algorithms and the best classifier was SVM. Diverse texture parameters distinguished significantly between fibroblastic and non-fibroblastic subtypes.	Demonstrated that machine learning classifiers based on radiomic features derived from T1C images, ADC, and FA maps were able to differentiate meningioma grades.
Morin et al. (19)	303	T1WI, T2WI, FLAIR, DWI/ADC, SPGR-T1CE	Grading, Local failure (LF), Overall survival (OS)	Manual	Shape, Histogram, Intensity, Texture, Wavelet filters	Supervised false-positive avoidance methodology	RF	ADC hypointensity (HR 5.56, 95% CI 2.01–16.7, P = .002) was corelated with WHO grade II/III meningioma, and low sphericity was associated both with increased LF (HR 2.0, 95% CI 1.1–3.5, P = .02) and worse OS (HR 2.94, 95% CI 1.47–5.56, P = .002).	Demonstrated that preoperative radiologic and radiomic features were able to predict tumor grade, LF, and OS in patients with meningioma.
Niu et al. (41)	241	CE-T1WI	Subtyping	Manual	Histogram, Texture, Form factor, GLCM, RLM	Spearman correlation analysis, t test analysis	LDA	Training: Accuracy=100%; Validation: Accuracy=94.2%. Both training model and validation method built on radiomic features showed high accuracy in the differentiation of meningothelial, fibrous and transitional subtypes.	Demonstrated that radiomics features and the combined statistical analysis were able to distinguish different meningioma subtypes preoperatively with satisfactory performance.
Li et al. (42)	67	T2-FLAIR, DWI, T1WI+C	Differential	Manual	Gray-level histogram, CM, RLM, Autoregressive model, Wavelet transform	‘Boruta’ Package in R software	SVM	Training: AUC=0.90. The performance of classifier based on the enhanced T1WI was significantly higher than that of T2-FLAIR-based or DWI-based classifiers in the differentiation between malignant hemangiopericytoma and angiomatous meningioma.	Demonstrated that SVM built on texture features extracted from T1C was capable of differentiating malignant hemangiopericytoma from angiomatous meningioma preoperatively.
Zhang et al. (43)	60	T2WI, DWI/ADC, T1C	Relapse Prediction	Automatic	Histogram, GLCM	RF	A binary decision tree	Training: Accuracy=0.90. The accuracy of radiomic model for prediction of P/R was higher than ADC values. The three most significant features contained in the final radiomics model were T1 max probability, T1 cluster shade, and ADC correlation.	Demonstrated that the radiomics approach was able to provide objective and important clinical information preoperatively for the cure of skull base meningiomas.
Tian et al. (44)	127	CE-TIWI T2WI	Differential	Manual	Histogram, GLCM	Mann–Whitney U test with the Benjamini–Hochberg method	Binary logistic regression analysis	Training: AUC=0.776, P = <0.001 The radiomic model built on the integrated quantitative parameters showed the best performance in differentiating craniopharyngioma from meningioma. These texture features were also significantly correlated with cystic alteration, which could be considered as diagnostic independent predictors.	Demonstrated that texture features could be used to distinguish craniopharyngioma from meningioma. The radiomic features were correlated with qualitative MR images features.
Zhang et al. (45)	1,728	T1C T2WI	Brain invasion	Manual	Shape, Histogram, Texture	LASSO	SVM	Training: AUC=0.857,。 Radiomics features were significantly correlated with brain invasion.	Demonstrated that the clinicoradiomic model was able to predict brain invasion in meningioma.

LoG, Laplacian of Gaussian; PCA, principal component analysis; FA, factor analysis; RF, random forest; AUC, area under the curve; CM, co-occurrence matrix; LR, logistic regression; NB, naive Bayes; SVM, support vector machine; Sub, Subtraction maps; LASSO, least absolute shrinkage and selection operator; SBS, sequential backward selection; LDA, linear discriminant analysis; CNN, convolutional neural networks; MC, multicentric; XGBOOST, eXtreme gradient boosting; MLP, multilayer perceptron; GBDT, gradient boosting decision tree; GLCM, gray-level co-occurrence matrix; RLM, run length matrix; P/R, progression/recurrence.

### Predicting Pathological Grade of Meningiomas

Tumor grade is a prerequisite to assess the necessity of a subsequent treatment of meningiomas. Currently, this kind of information, regarding the tumor grade, is available only after histopathologic inspection on tumor samples deriving from invasive biopsy or surgery (46). To achieve non-invasive pathological grading, the burgeoning development of radiomics has brought a new dawn in the preoperative grading prediction.

In the initial stages of radiomics analysis experimentation, multiple studies have explored the feasibility of various radiomic features in the prediction of pathological grade of meningiomas. The results showed that both conventional radiomic features, including the shape, histogram, texture, gray-level run length matrix, wavelet transform, and other higher-order statistics (19, 34–38, 40), and the DLR features (39) could predict the tumor grades. Yan et al. have identified two textural features based on the run length matrix and two shape-based features significantly related with the WHO grade II meningiomas; Similarly, in terms of the low grade meningiomas (WHO grade I), one textural feature based on run length matrix and one shape-based feature were selected (35). Zhu et al. have utilized up to 39 novel DLR features to distinguish high grade meningiomas (WHO grade II or III) from low grade ones (39). More detailed information of radiomic features applied in grade prediction and other aspects are summarized in **Table 2**.

**Table 2:** Summary of most useful set of radiomic features applied in grade prediction and other aspects.

Type Application		Morphology	Histogram	Texture	Deep learning
**Grade**	High	T1C_SD (34), T1C_GeoFv (35), T1C_GeoW4 (35), Roundness-of-FLAIR-shape (36)	T1C_HILAE (34), T1C_LILAE (34), T1C/ADC/FA_entropy (40)	T1C_RLN (34), T1C_Horzl_RLNonUni (35), T1C_S(2,2)SumOfSqs (35), Cluster-shades-of-FLAIR/T1CE-grey-level (36), DWI-ADC-grey-level-variability (36), FLAIR/T1CE-grey-level-energy (36), T1C/ADC/FA_dissimilarity (40), T1C/ADC/FA_RLN (40)	DLR from CNN (39)
	Low	T1C_GeoW5b (35), T1C_ Sphericity (19, 39)		T1C_WavEnHL_s-3 (35), T1C_ LGLRE (39)	
**Differentiation**	AM and HPC (42)			T1C_GLevNonU	
	MNG and CPG (44)		T1C_Skewness, T2_Skewness	T1C_GLCM-Contrast	
**Recurrence (** 43 **)**				T1C_GLCM_T1 maximum probability, T1C_GLCM_T1 cluster shade, ADC_GLCM_ADC correlation	
**Brain invasion (** 45 **)**		T1C_original_shape_maximum 2D diameter slice, T1C_original_shape_maximum 3D diameter		T2_lbp-3D-m2_glrlm_short run high grey level emphasis	

T1C_, contrast-enhanced T1-MRI; HILAE, High Intensity Large Area Emphasis; LILAE, Low Intensity Large Area Emphasis; SD, Spherical Disproportion; RLN, Run Length Non-uniformity; Horzl_RLNonUni, run length nonuniformity” with θ being 0°; S(2,2)SumOfSqs, “sum of squares” with θ being 45° and d being 2; GeoFv, vertical Feret’s diameter; GeoW4, GeoU1/GeoUw; GeoU1, the profile specific perimeter; GeoUw, the convex perimeter; DLR, deep learning features; CNN, convolutional neural networks; LGLRE, a sparse distribution of low gray-level values; GlevNonU, the grey-level nonuniformity; AM, angiomatous meningioma; HPC, haemangiopericytoma; MNG, meningioma; CPG, craniopharyngioma; GLCM, Grey-level co-occurrence matrix. For more explanations of these radiomic features please refer to each respective reference.

Notably, there are several common radiomic features across different studies regardless of their nonidentical nomenclatures (47). One radiomic feature is the sphericity, evaluating how a tumor is morphologically similar to a sphere (19, 36, 39), or spherical disproportion, rating the deviation of a lesion’s morphology from a sphere of the similar volume (34, 40). There were 5 studies explicitly demonstrating that high-grade meningiomas tend to have less sphericity than low grade meningiomas, in another word, high-grade meningiomas show more spherical disproportion than low grade ones (19, 34, 36, 39, 40). Moreover, one of these studies found that low sphericity was also associated with local recurrence and less favorable overall survival (19), which may imply that early intervention and shortening observation are warranted in meningiomas of low sphericity. The non-uniformity of the gray level or the run length matrix is another important radiomic feature, which is sensitive in reflecting the heterogeneity within the contoured area (34, 35, 38–40), such as the positive capsular enhancement, indistinguishable tumoral border, and heterogeneous tumor enhancement (46, 48). Because the fluctuance of parameters from second or higher order statistics revealed irregular changes in the gray pixels in aggressive meningiomas due to the intratumoral nonuniform structure tissue (49). Furthermore, it seems that diversified combinations of these features, such as a combination of radiomic features from different feature categories, multiple imaging sequences, heterogenous raw data or combined with qualitative imaging features or clinical data, could improve the performance of the classification models even if those improvements may not always be significant (19, 34, 37, 39).

In addition to radiomic features, the algorithm used in modeling is another critical factor affecting the performance of prediction (50). Since there has been no standardized guidance of algorithms selection yet, the selection usually depends on the preference and experience of analysts (32). At present, the classification methods presently applied for grade prediction include the random forest (RF) (19, 34, 37, 40), logistic regression (LR) (35, 36), naïve Bayes (NB) (35), support vector machine (SVM) (35, 37, 38, 40), eXtreme gradient boosting (XGBOOST) (37), multilayer perceptron (MLP) (37), and linear discriminant analysis (LDA) (38, 39) (**Table 3**). Among these various algorithms, numerically, the best performance of prediction was achieved in a tree-based classification algorithm, XGBOOST, which based on a combination of features derived from multiple MRI sequences and yielded a high AUC of 0.97, a sensitivity of 1.0 and a specificity of 0.97 (37). While the most widely used algorithms are the RF and SVM, the RF is an ensemble method that calculates multiple decision tree-based classifiers containing several identically distributed random independent vectors (37, 51), whereas the SVM is a non-linear classifier that iteratively constructs a hyperplane or high-dimensional feature space consisting of a series of hyperplanes that separates different classes (52, 53). There have been two studies comparing different performances of the RF and SVM, Hamerla et al. have built four different classification models, including the RF, XGBOOST, SVM, and MLP, based on same radiomic features; Their results demonstrated both RF and SVM had same AUC of 0.93 (37). However, in the study of Park et al., the SVM have shown to have a better predicting performance with an AUC of 0.86 comparing to 0.84 in the RF (40). Whereas it was contradictory in the prognosis prediction superiority between the RF and SVM in a study of lung cancer (54). Actually, the RF has a number of advantages, such as its totally non-parametric property, so that it can be used given the existence of collinearities among features (55). Furthermore, overfitting is less of a concern compared with other machine-learning methods (51). Therefore, all these characteristics make RF especially suitable for high-dimensional data analyses as radiomics, where it is impractical to strictly control all features (56).

**Table 3:** Summary of commonly used algorithms along with their performance metrics.

Algorithm	Description	Performance metrics
**Random forest**	An ensemble method that calculates multiple decision tree-based classifiers containing several identically distributed random independent vectors.	AUC=0.93 Sensitivity=0.90 Specificity=0.97 (37)
**Support vector machine**	A non-linear classifier that iteratively constructs a hyperplane or high-dimensional feature space consisting of a series of hyperplanes that separates different classes.	AUC=0.93 Sensitivity=0.95 Specificity=0.94 (37)
**eXtreme gradient boosting**	A tree-based classification algorithm where an ensemble of decision trees is built.	AUC=0.97 Sensitivity=1.00 Specificity=0.97 (37)
**Multilayer perceptron**	A feed-forward deep artificial neural network.	AUC=0.88 Sensitivity=0.95 Specificity=0.87 (37)
**Linear discriminate analysis**	A linear classifier, consisting of the shape of the decision boundary of straight line in the first case and straight line in second.	AUC=0.934 Accuracy= 0.756 (38)
**Logistic regression**	A kind of multiple regression method to analyze the relationship between a binary outcome or categorical outcome and multiple influencing factors.	AUC=0.85 Accuracy=0.89 Sensitivity=0.67 Specificity=0.94 (35)
**Naive Bayes**	Acyclic directed graphs, in which each node of the graph represents a variable and each arc is a direct probabilistic relationship between the variables.	AUC=0.91 Accuracy=0.89 Sensitivity=0.76 Specificity=0.92 (35)
**Convolutional neural network**	Deep learning networks comprising hundreds of self-learning units had advantages in quantifying the prognostic features that could not be manually defined.	AUC=0.811 Sensitivity=0.769 Specificity=0.898 (39)

Besides, there was also a comparison study between the SVM and LDA, in which Chen et al. had shown that a LDA-based model displayed an AUC of 0.934 in predicting the WHO grade I meningiomas higher than that of 0.845 in a SVM-based model (38). Both of them are regarded as the top pattern recognition technology, functioning obeying to two different working principles (57). Whereas in the non-linear attribute of the SVM, the LDA is a linear classifier which means the shape of the decision boundary of LDA is a straight line, or a plane different from that of curved lines, or a surface in SVM (57). Additionally, when comparing the classification algorithms, their comparison also contained the selection method, and their results indicated that the modeling algorithms may weigh more than that used in feature selection processes in the aspect of increasing the diagnostic performances (38). As for the LR and NB, it is imperative to pay attention to their inherent limitations, including the independence assumption to features of the LR and the request for feature discretization in the NB (32). The novel machine learning method, MLP, which had also been utilized in modeling, though not the best, exhibited a predicting performance of 0.88 (37). Moreover, another deep learning method, convolutional neural network has been implemented in the process of feature extraction; Instead of modeling, it provided a better predictive performance than the hand-crafted features (39).

### Other Applications in Meningiomas

Radiomics analysis has also shown to be predictive in other aspects of meningiomas, like subtypes identification, differential diagnosis, recurrence prediction and brain invasion. Niu et al. have extracted 385 radiomic features from the T1C images of 241 patients and built a Fisher discriminant analysis model which successfully distinguished subtypes of meningothelial, fibrous, and transitional meningiomas yielding a perfect accuracy of 100% with an as high accuracy of the validation model as 94.2% (41). Another study also reported that there were significant differences in various texture features derived from the T1C, ADC, and FA parameters between the fibroblastic and nonfibroblastic pathological subtypes, without establishing a radiomic model (40).

Regarding the differential diagnosis, a study has constructed three SVM classifiers based on texture features respectively derived from the T2-FLAIR, DWI and enhanced T1WI sequences to compare their capacities in differentiating malignant hemangiopericytomas from angiomatous meningiomas. Their results indicated that the enhanced T1WI-based classifier (AUC = 0.90) had significantly better performance than the T2-FLAIR-based and DWI-based classifiers (42). Specifically, a recent study has selected three independent imaging predictors, including skewness, contrast on the contrast-enhanced images, and skewness derived from the T2WI to distinguish craniopharyngiomas and meningiomas, and the binary logistic regression model built on the three integrated radiomic features achieved an AUC of 0.776. Moreover, it was also discovered that these texture features were significantly related with the cystic alteration which was found as the only independent diagnostic predictor in qualitative imaging features in their research (44).

Regarding the relapse prediction, a study has extracted 99 radiomic features from the T2WI, DWI, and T1C and has filtered the three most significant parameters as the T1 max probability, T1 cluster shade, and ADC correlation in predicting the recurrence of skull base meningiomas. The accuracy of predicting recurrence in their binary decision tree model, which was founded on these three features, was 0.90 higher than that of the other model based on ADC values (43). Besides, there was a study with a relatively large multi-institution sample size, composed of 303 patients revealed that the low sphericity was associated with not only the increased local recurrence but also worse overall survival; The integrated RF model combining radiomic, radiologic, and clinical features showed an AUC of 0.75 and 0.78 in predicting local recurrence and overall survival, respectively (19).

More recently, a multicenter study has shown that radiomic features have the potential of preoperatively predicting brain invasion in meningioma (45). They have built a SVM model derived from the T1C and T2 MRI sequences and yielded an AUC of 0.819. What’s more, the clinicoradiomic model integrating radiomic features and sex information exhibited the best predictive performance (AUC=0.857).

## Limitations of Radiomics Analysis for Meningiomas

As radiomics is still in its initial phase of application in meningiomas, there are still many drawbacks to overcome in its whole process. Currently, most radiomics studies in meningiomas were designed as unicentric and retrospective studies which can lead to selection bias (19, 36–40, 42, 43, 58). Another prominent issue is the lack of high-quality raw data, which manifested mainly as the significant heterogeneity of patient cohorts or imaging data and small sample sizes (28, 29). A wide variety in the overall staging of patients may be a confounding factor since staging itself is usually of prognostic significance (28). The heterogeneity of the original data can introduce changes that may be not due to the underlying biological effects (29). Small sample sizes can increase both statistical error rates and the risk of overfitting (28). There is not only a lack of original data, also an insufficient utilization, as quite many studies only used a portion of the imaging data; For example, in some study, only the enhancing sequences were selected rather than all sequences of MRI (40), or features were extracted from a series of consecutive slices instead of all slices (39).

Beyond these limitations in designing and imaging acquisition, there are some considerable problems in the rest of the procedure, especially in the segmentation process (29). To date, most meningiomas radiomics studies have based on manual segmentation, which can lead to greater inter-observer variations. Although the assistance of segmentation software or DLR can reduce the difference, whether the timely update of these assistive tools can be achieved is another challenge. Besides, the time cost of manual delineation is also an important consideration especially when an operator is facing hundreds of patients simultaneously (7). The conventional manual feature extraction relies on predefined algorithms designated for specific imaging characteristics. As different extraction techniques and software were chosen, different results of features were generated which apparently could lead to bias in the results (7, 59). All these changes can impact on the reproducibility of the features, which unfortunately, directly determines the generalization of the research conclusion (7, 28). Consequently, with the advanced imaging modalities continuously emerging, the need of autonomic learning algorithms with the capacity of handling integrated multiparametric imaging data is increasingly urgent (60). Likewise, at the final stage of the radiomics workflow, the requirement for constantly improved models is also increasing. Lots of studies have to face the problems from over-simple correlation analyses, such as contradictory conclusions of similar situations from different researchers or the risk of overfitting or underfitting (7), the insufficient interpretation of the data, and the lack of machine learning or other advanced statistical analysis methods (38, 43).

Above all, all the variations in the aforementioned steps call for the standardization or the guideline of the detailed implementation procedure of the radiomics workflow in different situations. In the meantime, the boundary of data sharing among different institutions is still vague, and it is necessary to establish and improve relevant laws and regulations.

## Future Perspectives of Radiomics Analysis for Meningioma

As the capability and potential of radiomics are increasingly revealed, many different aspects still merit future developments.

### Curation of Big Data

The curation of big data plays a prerequisite role in the efficacy and efficiency of radiomics. Generally, from a biopsychosocial view, big data related to radiomics should not only include imaging data but also involve demographics and social networks. This seemingly insignificant information reflects the compliance of meningioma patients under surveillance having a prognostic value, which should be taken into consideration during the model building, especially in meningiomas suspicious of malignancy. Furthermore, radiomics has shown its application in the prediction of genomics, proteomics or other biology–omics. The integration of those different datasets also places requirements of curation. Specifically, the annotations of imaging data currently often do not use a standard lexicon, this hinders efficient utilization of data (29). Also, given the fact that the majority of present radiomics studies are retrospective, prospectively collected imaging data is in urgent need. This situation requires radiologists and clinicians to actively participate in the beginning period of data gathering rather than merely in later analysis.

### DLR Analysis

DLR analysis has shown its powerful advantages compared to the conventional radiomics demonstrating as automatic operation, full exploitation of data, free of manual variance, low labor-consuming, etc. However, there is still a range of gaps for the DLR to overcome. The DLR eagers to embrace big datasets equipped with millions of images for the requirement of training, because the high-quality training data is directly related with better performance of the DLR (61). The cost of the computational infrastructure of the DLR is another bottleneck that appeals to data scientists to contribute more efforts (62). Interestingly, on one hand, scientists put great expectations on the future of the DLR, on the other hand, they have not completely understood how deep learning works yet, namely as the fear of ‘black box’ (63). In a word, DLR is a double-edged sword which enlightens us with its intelligence except for careful trust.

### Comprehensive Interdisciplinary Cooperation

The development of radiomics owes to the devotion of both clinical and technical investigators. This new discipline shows a continuous progression, together with a mutual competition between those two sides of the research. Indeed, conventionally separated, these two aspects of research are used to analyze problems from their own perspective, overcoming the benefits of the other. This condition may explain the existence of simple correlations between imaging features and clinical significance, regardless of methodological disadvantages, or the pursuit of novel methodology while neglecting the clinical significance (7). Indeed, the thrive of radiomics does not only rely on balancing the interdisciplinary contradiction, it also demands the expansion of the collaboration, including the introduction of data source, enhancement in data sharing, renewing, and accessibility.

## Conclusion

Radiomics analysis for meningiomas is a promising new area of research based on the development of computational advances. The current correlation is mainly between the imaging phenotypes and meningioma grades. With overcoming limitations in the process of the radiomics analysis, there will be a vast expansion of its applications in meningiomas, varying from risk stratification, to precise diagnosis, prognosis, and therapy.

## Author Contributions

Conception or design of the work: HG and TX. Drafting the paper: HG, XuZ, and PR. Revising it critically for important intellectual content: XiZ and TX. Provide approval for publication of the content. All authors contributed to the article and approved the submitted version.

## Funding

This work was financially supported by the National Natural Science Foundation of China (No.81472354, TX), Shanghai Outstanding Young Physician Program (TX).

## Conflict of Interest

The authors declare that the research was conducted in the absence of any commercial or financial relationships that could be construed as a potential conflict of interest.
